# Isolation and Polyphasic Characterization of *Desulfuromonas versatilis* sp. Nov., an Electrogenic Bacteria Capable of Versatile Metabolism Isolated from a Graphene Oxide-Reducing Enrichment Culture

**DOI:** 10.3390/microorganisms9091953

**Published:** 2021-09-14

**Authors:** Li Xie, Naoko Yoshida, Shun’ichi Ishii, Lingyu Meng

**Affiliations:** 1 Department of Civil Engineering, Nagoya Institute of Technology (Nitech), Nagoya 466-8555, Aichi, Japan; xie705049124@126.com (L.X.); meng.lingyu@nitech.ac.jp (L.M.); 2 Institute for Extra-Cutting-Edge Science and Technology Avant-Garde Research (X-Star), Japan Agency for Marine-Earth Science and Technology (JAMSTEC), Yokosuka 237-0061, Kanagawa, Japan; sishii@jamstec.go.jp

**Keywords:** *Desulfuromonas*, electrogenic bacteria, nitrate respiration, graphene oxide

## Abstract

In this study, a novel electrogenic bacterium denoted as strain NIT-T3 of the genus *Desulfuromonas* was isolated from a graphene-oxide-reducing enrichment culture that was originally obtained from a mixture of seawater and coastal sand. Strain NIT-T3 utilized hydrogen and various organic acids as electron donors and exhibited respiration using electrodes, ferric iron, nitrate, and elemental sulfur. The strain contained C16:1ω7c, C16:0, and C15:0 as major fatty acids and MK-8, 9, and 7 as the major respiratory quinones. Strain NIT-T3 contained four 16S rRNA genes and showed 95.7% similarity to *Desulfuromonas* *michiganensis* BB1^T^, the closest relative. The genome was 4.7 Mbp in size and encoded 76 putative *c*-type cytochromes, which included 6 unique *c*-type cytochromes (<40% identity) compared to those in the database. Based on the physiological and genetic uniqueness, and wide metabolic capability, strain NIT-T3 is proposed as a type strain of ‘*Desulfuromonas versatilis*’ sp. nov.

## 1. Introduction

Anaerobic and extracellular electron-transferring (EET) bacteria are ubiquitously involved in the redox flow via solid conductors. *Geobacter* and *Shewanella* have been extensively studied for their functional roles in terrestrial and marine subsurface, whose anoxic environments are deficient in soluble electron acceptors. The unique metabolism of such bacteria has been applied in bioelectrochemical systems (BESs) to be used for wastewater treatment [1], production of renewable energy and value-added products [2], and bioremediation [3]. Both culture-dependent and -independent studies have revealed the presence and functional role of electrogenic microbes other than *Geobacter* and *Shewanella* [4]. Various factors, such as availability of the electron donor [5], the electric potential of a solid conductor [6], and the origin, affect the microbial composition in the system. Additionally, the surface chemistry of electrodes provides selective pressure during the early growth of the biofilm [7].

The genus *Desulfuromonas* has received much attention as a common electrogenic bacteria present in BESs [8,9,10]. *Desulfuromonas* species are found in natural environments, such as aquifers [11], sediment cores [12], and terrestrial mud volcanoes [13], suggesting a wide distribution. The genus *Desulfuromonas* consists of anaerobic chemoheterotrophs and was first proposed after the isolation of an elemental sulfur-reducing bacterium, *Desulfuromonas acetoxidans* DSM 684^T^, from anaerobic sulfide-containing marine mud [14]. At present, *D*. *acetoxidans* DSM 684^T^ and seven other valid species have been proposed. *D.* *acetexigens* 2873^T^ [15], *Desulfuromonas* *palmitatis* SDBY1^T^ [16], *Desulfuromonas* *thiophila* NZ27^T^ [17], *Desulfuromonas* *chloroethenica* TT4B^T^ [18], *Desulfuromonas* *michiganensis* BB1^T^ [19], *Desulfuromonas svalbardensis* 112^T^ [20], and *Desulfuromonas* *carbonis* ICBM^T^ [21] have been isolated from anoxic freshwater sediments, marine sediment, freshwater mud, freshwater sediment, pristine river sediment, Arctic marine sediments, and coal-bed water, respectively. Recently, additional strains including *Desulfuromonas* sp. TF [22], *Desulfuromonas* sp. AOP6 [23], *Desulfuromonas* sp. TZ1 [24], and ‘*Desulfuromonas soudanensis*’ WTL [25] have been isolated from tidal flat sediment, sub-seafloor sediment, marine sediments, and anoxic deep subsurface brine, respectively.

All valid species of *Desulfuromonas* grow anaerobically and reduce sulfur, and exhibit iron respiration coupled with acetate oxidation; however, they are unable to respire using soluble nitrogenous and sulfuric compounds. The respiration specific to solid minerals and the presence of *Desulfuromonas* species in BESs suggest the substantial involvement of *Desulfuromonas* in electrode-driven metabolism, although such activity has been rarely proven in pure cultures. None of the valid species, except for two strains including *Desulfuromonas* sp. TZ1 [24] and ‘*D. soudanensis’* WTL [25], generate an electric current in pure cultures, and both have been isolated from electrodes set up in environments, such as marine sediment and Soudan mine, respectively. These results suggest that *Desulfuromonas* species play substantial roles similar to those of two representative electrogenic genera, *Geobacter* and *Shewanella,* in microbial elemental cycling on solid conductors, especially in marine sediments.

Presently, the genomes of two strains, *D. soudanensis* WTL [25] and *Desulfuromonas* sp. AOP6 [23], have been published, and the genome of another strain, DDH964, is unpublished but uploaded on the National Center for Biotechnology Information (NCBI) database under the accession number CPO15080. The genomes are 1.64–4.40 Mb in size, contain 37.9–65.9% of broad G + C content, and have 2181–3924 coding sequences (CDSs). All three genomes encode a complete TCA cycle, a non-oxidative pentose phosphate pathway, Embden-Meyerhof-Parnas glycolysis/gluconeogenesis, and abundant CDSs associated with *c*-type cytochrome biosynthesis. The number of putative multiheme *c*-type cytochromes ranges from 37–44, and the number is comparable to those in the genera *Geobacter* and *Shewanella*. Additionally, genes involved in the biosynthesis of type IV pili, known to be involved in the formation of conductive biofilm [26], are commonly present in the three genomes.

In this study, a novel electrogenic bacterium of the genus *Desulfuromonas*, ‘*Desulfuromonas versatilis*’ NIT-T3 was isolated from an enrichment culture of graphene oxide-reducing bacteria (GORB) that were initially obtained from a mixture of seawater and coastal sand [27]. GORB have been applied in the formation of hydrogel electrodes that generate electricity using synthetic medium [28], soil [29], and wastewater [30,31,32]. Physiological and genomic analysis of strain NIT-T3 revealed versatile metabolism, and the findings expand the understanding of the metabolism in the genus *Desulfuromonas* and its ecology in natural and artificial environments.

## 2. Materials and Methods

### 2.1. Isolation and Growth Conditions

Strain NIT-T3 was isolated from an enrichment culture of GORB (CS culture) that was obtained from a mixture of seawater and coastal sand, as described previously [27]. A DS-basal medium used for the isolation and growth of the strain contains 20 g/L NaCl, 0.3 g/L KCl, 0.5 g/L NH_4_Cl, 0.1 g/L CaCl_2_·2H_2_O, 4 g/L MgCl_2_·6H_2_O, 0.6 g/L KH_2_PO_4_, 2.5 g/L NaHCO_3_, 1 mL/L SL-10, 1 mL/L Se/W solution, vitamin-solution, and 0.2 mg/L resazurin and the basal medium was prepared anaerobically under flashing N_2_:CO_2_ (80:20, *v*/*v*) [28]. The strain NIT-T3 was isolated from an anaerobic DS-AQDS agar plate which DS-basal medium supplemented with 10 mM acetate, 5.0 mM anthraquinone-2,6-disulfonate (AQDS), 1 mM Na_2_SO_4_, and 1.5% agarose. After 7–14 d of incubation at 28 °C, electrochemically active colonies showed orange halos, which was the color of the reduced form of AQDS. The colony culture was then purified via repeated agar-shake cultivation using DS-AF agar plate, DS-basal medium supplemented with 10 mM acetate, 0.1% yeast extract, 1.5 mM Na_2_S, 5.0 mM fumarate, and 1.5% agarose. The purified culture was then phylogenetically identified based on the 16S rRNA gene sequence amplified from the cell lysate [33] and named strain NIT-T3. Strain NIT-T3 was routinely cultured using the liquid DS-AF medium. In total, 7–14 d of anaerobic cultivation at 28 °C was sufficient to achieve full growth. 

### 2.2. Morphological, Physiological, and Biochemical Analyses

The morphology of strain NIT-T3 was evaluated using field-emission scanning electron microscopy (JSM-7800F; JEOL Ltd., Tokyo, Japan) operating at 1.0 kV; spore-forming ability and Gram staining nature were determined via optical microscopy, as described previously [34]. Motility was determined using the hanging drop method [34]. The effect of NaCl on cell activity was evaluated by monitoring growth in DS-AF medium supplemented with 0–8.0% (*w*/*v*) NaCl. The effect of pH was also determined using a bicarbonate-free medium adjusted to a pH ranging from 4.8 to 8.4 using sodium bicarbonate and by adjusting the CO_2_ concentration in the headspace gas. Temperatures ranging from 4 °C to 40 °C with approximate intervals of 5 °C were applied to determine the effect on cell growth.

Formate, acetate, butyrate, lactate, pyruvate, succinate, malate, isopropanol, glucose, glycerol, isobutyrate, caproate, benzoate, phenol, methanol, ethanol, butanol, and fructose at 10 mM, and 0.5 g/L peptone and yeast extract were tested as potential electron donors during nitrate reduction. Potential electron acceptors were evaluated by observing growth and detecting the oxidation of 5.0 mM acetate in the presence of 10 mM fumarate, 5 mM nitrate, 10 mM sulfate, 10 mM thiosulfate, 5.0 mM AQDS, and 10 mM malate. Production of electric current by the strain NIT-T3 was evaluated via electrochemical cultivation using a graphite plate inoculated with NIT-T3, as described previously [35].

### 2.3. Chemotaxonomic Analysis

The cellular fatty acid composition and isoprenoid quinones present in NIT-T3 were investigated by Techno Suruga Laboratory Co., Ltd. (Shizuoka, Japan). Isoprenoid quinones were extracted, as described by Tamaoka et al. [36]. Cellular fatty acids were analyzed using cells cultured in liquid DS-AF medium at 28 °C for 14 d. The fatty acid profile was analyzed using the Sherlock Microbial Identification System version 6.0 (MIDI) using the TSBA6 database.

### 2.4. Genetic Characterization

Genomic DNA was extracted from strain NIT-T3, as described previously [37]. Sequencing was performed using a combination of the Illumina Miseq and Nanopore MinION. In total, 1.8 M reads (1.02 Gbp) of Illumina paired-end reads (150 × 2) and 0.37 M Nanopore reads (1.76 Gbp) were subjected to error removal using Short Read Manager and assembled using Unicycler-0.4.7. The complete genome of NIT-T3 was successfully determined, and gene prediction and genome annotation were performed using DFAST [38]. Comparison of the genes between NIT-T3 and other *Desulfuromonas* species was based on bidirectional best hits at 40% identity and 80% query coverage using SEED Viewer version 2.0 [39] and basic local alignment search tool in the NCBI database. The sequence of the NIT-T3 genome was deposited in the DNA Databank of Japan/GenBank under the accession number AP024355.

The 16S rRNA gene sequences from all publicly available *Desulfuromonas*, *Desulfuromusa*, *Geobacter*, *Geopsychrobacter*, and *Pelobacter* genomes were downloaded from NCBI. A phylogenetic tree based on 16S rRNA gene sequences of NIT-T3 and other members of the family *Desulfuromonadaceae* was generated using MEGA X based on the neighbor-joining method [40].

## 3. Results

### 3.1. Isolation of NIT-T3

**Figure 1:**
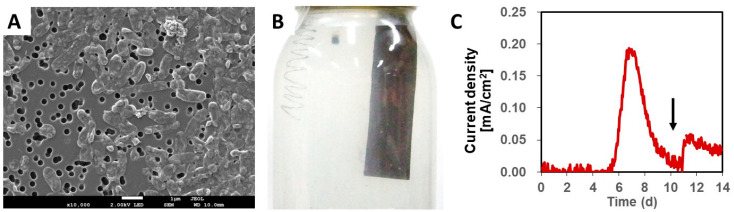
Morphology and electrogenic properties of strain NIT-T3. (**A**) Scanning electron microscopic image of strain NIT-T3. The white bar indicates 1 μm of length. (**B**) Growth of a biofilm on an electrode in an electrochemical culture. (**C**) Electric current production by strain NIT-T3. The arrow in panel C indicates a spike of acetate addition.

Anaerobic cultivation of AQDS-supplemented agar plates inoculated with the TGOA enrichment culture resulted in the formation of colonies that showed a change in color from colorless to orange. This suggested the ability of the colony to reduce AQDS and utilize it as an extracellular electron acceptor. A single colony was picked from the agar culture, purified via repeated agar-shake cultivation, and then re-cultivated in liquid DS-SF medium. Finally, based on the uniformity in microscopic morphology and 16S rRNA gene sequences, a liquid culture was selected and further purified by repeating the agar cultivation step. Cells of strain NIT-T3 were Gram-negative, non-spore-forming, rod-shaped, and approximately 0.5 μm in width and 1.5 μm in length (Figure 1A).

The strain NIT-T3 produced an electric current on a graphite electrode in the presence of acetate and simultaneously generated a thin biofilm on the electrode surface (Figure 1B). The current in the electrochemical cultivation medium was rapidly generated and the maximum level was detected in the range of 0.18 to 0.19 mA/cm^2^ on days 6 and 7 (Figure 1C). Electric current production decreased gradually with time; however, it increased immediately after adding acetate to the media. These results indicated that strain NIT-T3 grows by coupling EET to electrode with acetate-oxidation.

### 3.2. Phylogenetic Identification Based on 16S rRNA Sequencing

**Figure 2:**
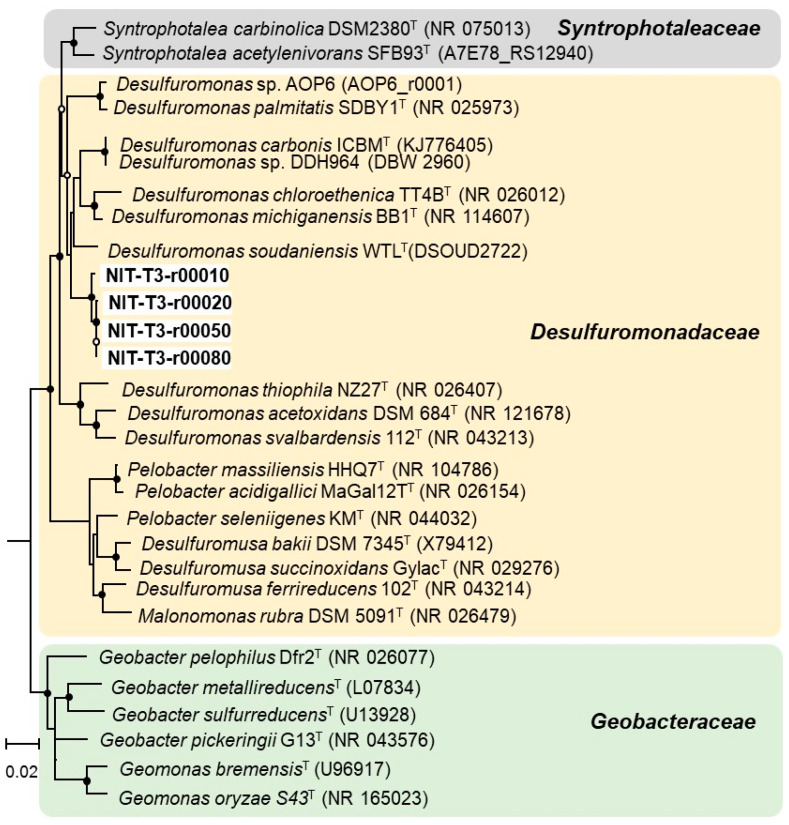
Phylogenetic tree generated using 16S rRNA gene sequences of members of Desulfuromonadales. Closed and open circles indicate bootstrap > 80% and 60%, respectively. GenBank accession numbers are stated in parentheses.

Strain NIT-T3 was found to contain four 16S rRNA operons (*rrn*1-4); one of them showed 95.5–95.8% similarity to the three *Desulfuromonas* strains. The copy number was higher than that in *Desulfuromonas* strains containing two copies, and equal to that in four *Geobacter* and two *Pelobacter* strains: *G. bemidjiensis*, *G. bremensis*, *P. acetylenicus*, and *P. propionicus*. The 16S rRNA gene-based phylogenetic tree revealed that all four *rrn*s formed a cluster with sequences of five strains of the genus *Desulfuromonas* of the family *Desulfuromonadaceae* (Figure 2).

Strain NIT-T3 showed only 95.7% closest similarity based on 16S rRNA gene sequences to that of *D. michiganensis* BB1^T^. Similarities with seven other species of the genus *Desulfuromonas* ranged from 92.9% to 95.4%. The 16S rRNA gene sequence similarity of strain NIT-T3 to members of the genera *Pelobacter, Desulfuromusa,* and *Geobacter* were 89.7–94.9%, 90.7–90.9%, and 90.6–91.6%, respectively. According to the cut-off values of 98.2–99.0% [41], and 98.65% similarity among single species [42], the strain NIT-T3 may be proposed as a strain of a novel species of the genus *Desulfuromonas.*

Strain NIT-T3 grew at 10–35 °C (optimum, 25 °C), pH 6.4–8.4 (optimum pH 6.8–7.1), and tolerated 0.05–3% NaCl (optimum, 0.2–1%) (Table 1). Growth was completely inhibited at concentrations of ≥3% NaCl. Strain NIT-T3 could metabolize hydrogen, formate, acetate, lactate, pyruvate, succinate, malate, peptone, isopropanol, and yeast extract in the presence of nitrate (Table 1). Lactate, pyruvate, succinate, and the combination of H_2_ and acetate resulted in optimal growth together with nitrate. No growth occurred in the presence of butyrate, glucose, glycerol, ethanol, isobutyrate, caproate, benzoate, phenol, methanol, butanol, or fructose. Cells did not show any apparent movement on a slide, suggesting that the strain NIT-T3 was non-motile.

### 3.3. Physiological and Biochemical Characterization

**Figure 3:**
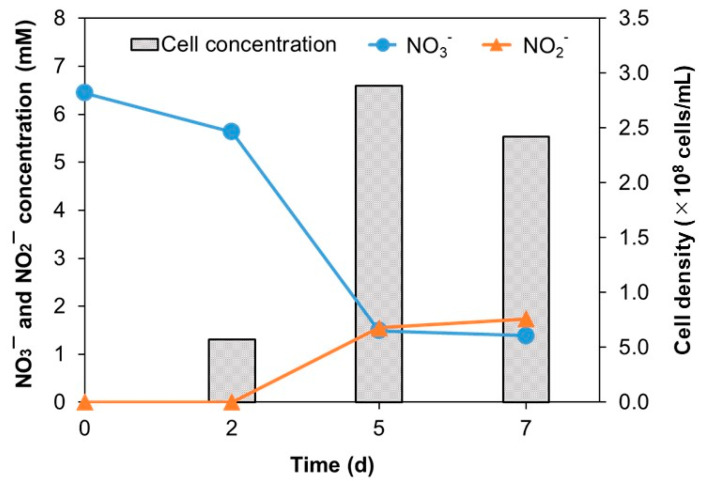
Changes in the NO_3_^−^ and NO_2_^−^ concentrations and NIT-T3 cell densities in the culture supplemented with nitrate.

Similar to most *Desulfuromonas* species, strain NIT-T3 showed respiration using sulfur and ferric iron coupled with acetate oxidation. Contrary to the lack of nitrate respiration in other *Desulfuromonas* members, strain NIT-T3 exhibited nitrate respiration (Figure 3). The cell density increased from 5.7 × 10^7^ to 2.4 × 10^8^ cells/mL with a reduction of 5.1 mM of nitrate within 7 d of incubation. Meanwhile, a maximum concentration of 1.7 mM of nitrite was produced in the medium. The NIT-T3 did not grow in the culture supplemented with nitrite. These results indicated the ability of strain NIT-T3 to grow on nitrate, while reducing nitrate to nitrite. The imbalance of the spiked nitrate and produced nitrite suggested the assimilative utilization of nitrite as nitrogen source.

### 3.4. Chemotaxonomic Characterization

The major fatty acids identified in strain NIT-T3 were C_16:1_ω7*c* (26%), C_16:0_ (18%), and C_15:0_ (13%) (Table 1). The fatty acid profile of *Desulfuromonas* species is available for only two strains, *D. svalbardensis* 112^T^ [20] and *D. carbonis* ICBM^T^ [21]; these two strains contain the three major fatty acids found in strain NIT-T3. However, the proportions differ among the three strains. The most abundant fatty acid is C_16:0_ (approximately 40%) in strains 112^T^ and ICBM^T^, and C_15:0_ in ICBMT is detected in minor amounts (0.1%). The presence of C_16:1_ω7*c* and C_16:0_ as main cellular fatty acids was common in most members of *Geobacter* [43,44,45,46,47] rather than in only *Desulfuromonas*.

The major respiratory quinones in NIT-T3 were identified as MK-8 (93%), and other menaquinones including MK-7 (1.9%) and MK-9 (5.3%) were detected. This result is consistent with the fact that MK-8 is a typical respiratory quinone present in the genus *Geobacter* [43,44,47,48], although the menaquinone profile data of other species of the genus *Desulfuromonas* are not available.

### 3.5. General Genomic Features

**Figure 4:**
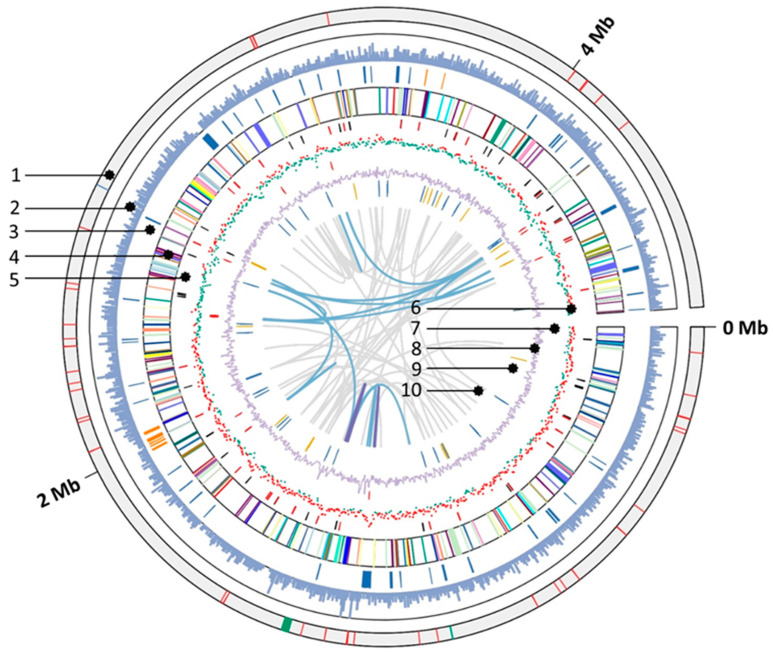
Features of the complete genome of ‘*Desulfuromonas versatilis*’ NIT-T3. Circular representation of the genome was generated using TBtools v.1.082 [49]. Rings numbered from the outside to inside are: 1, location of tRNA (red), transfer messenger RNA (blue), and rRNA (green); 2, gene density; 3, *c*-type cytochromes (blue), and type IV pili (orange); 4, protein coding sequences colored based on KEGG category; 5, putative sensor histidine kinases (red) and response regulators (black); 6, G + C skew (red, positive; green, negative) approximated using GenSkew (http://genskew.csb.univie.ac.at); 7, transposase (red) and phage integrase (black); 8, G + C content; 9, genes unique to NIT-T3 (orange, annotated; blue, unannotated) compared to other *Desulfuromonas* spp; 10, links showing repetitive sequence ≥ 95% identity (cyan, > 500 bp; purple, >2 kbp). KEGG, Kyoto Encyclopedia of Genes and Genomes.

NIT-T3 contained a single 4,656,376 bp circular chromosome that encodes 4119 protein-coding sequences (CDS), 60 transfer RNAs, 1 transfer-messenger RNA, and 10 rRNAs. The G + C content in strain NIT-T3 was approximately 63.1%, which is similar to that of *D. acetexigens* 2873^T^ and *D. acetoxidans* (Table 1). The genome map is shown in Figure 4. Regarding energy conversion, NIT-T3 had CDSs to metabolize hydrogen, lactate, pyruvate, and other organic acids of the TCA cycle intermediates (fumarate, succinate, malate), and complete TCA cycle. NIT-T3 also contained a full set of genes associated with glycolysis similar to the other two strains; however, the bacteria cannot utilize glucose due to the lack of glucose transporters [25]. A gene encoding nitrate reductase (*NarB*) was found which oxidizes quinol and reduces nitrate to nitrite. The reductive acetyl-CoA pathway and reductive TCA cycle lacked certain genes, suggesting an inability of the strain to fix carbon. 

### 3.6. Putative c-Type Cytochromes

**Figure 5:**
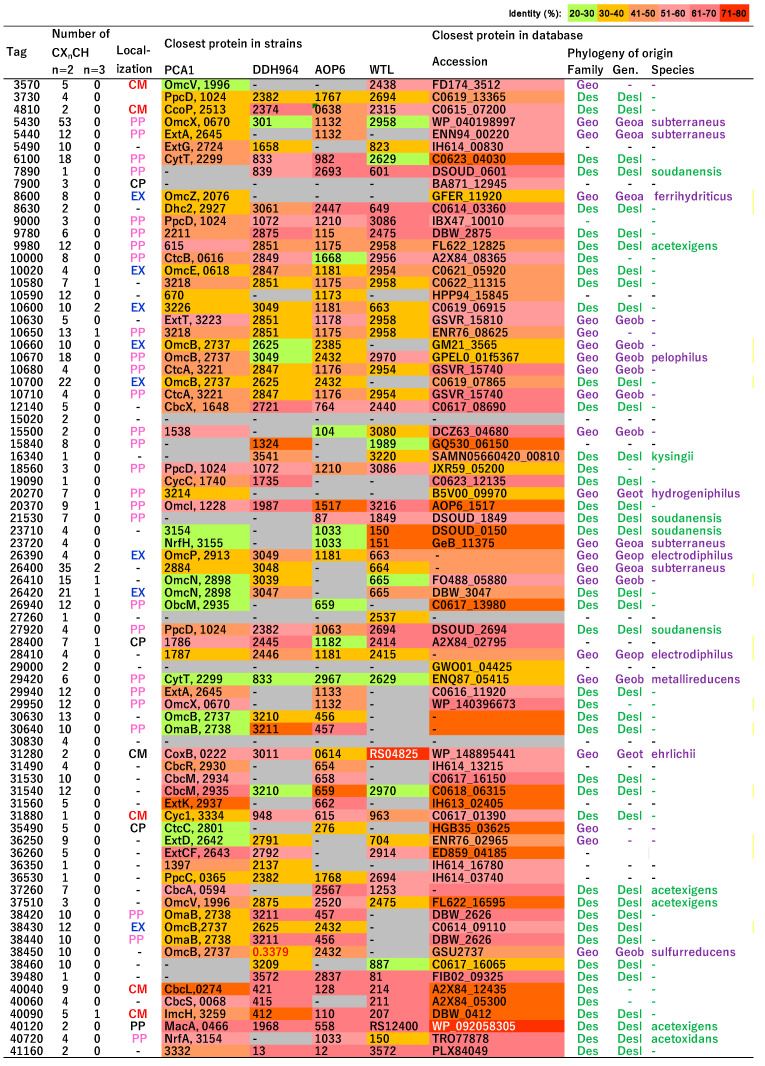
List of putative *c*-type cytochrome C proteins present in the NIT-T3 genome. Tag indicates the locus tag of the coding sequence encoding *c*-type cytochromes in the genome. CM, cytoplasmic membrane; PP, periplasm; EX, extracellular; (-), unknown. The number and color scale for the closest protein in the strain indicates the locus tag and amino acid identity (%), respectively. Geo, *Geobacteraceae*; Geoa, *Geoalkalibacter*; Geob, *Geobacter;* Geop; *Geopsychrobacter;* Geot*, Geothermobacter;* Syn, *Syntrophotaleaceae;* Synt, *Syntrophotalea;* Des, *Desulfuromonadaceae;* Desl, *Desulfuromonas*.

NIT-T3 possessed 79 putative *c*-type cytochromes. The CDSs with C(X)_n_CH, *n* = 2–4, were first screened and then identified as *c*-type cytochromes either based on the presence of a conserved domain of *c*-type cytochromes in the pfam and TIGR databases (e-value = <0.01 cutoff) or showing an e-value < 10^−10^ in the pairwise alignment with *c*-type cytochromes of strain PCA. Among them, 61 were homologs of *c*-type cytochromes in *Desulfuromonas* strains (Figure 5, ≥40% amino acid identity (AAI) in the 759–92 AAs of the HIPER scoring region) and 38 were homologs of those in *Geobacter sulfurreducens* PCA, a well-characterized model of iron-reducing bacterium. NIT-T3 contained a relatively larger number of *c*-type cytochromes than other *Desulfuromonas* strains in the range from 37 to 44. Based on PROSITE prediction [50], most of the *c*-type cytochromes were present in the periplasmic (28), extracellular (7), and cytoplasmic membranes (6), whereas a few cytochromes were present in the cytoplasm (3). The number of heme-binding motifs varied, and ranged from 1 to 53.

**Figure 6:**
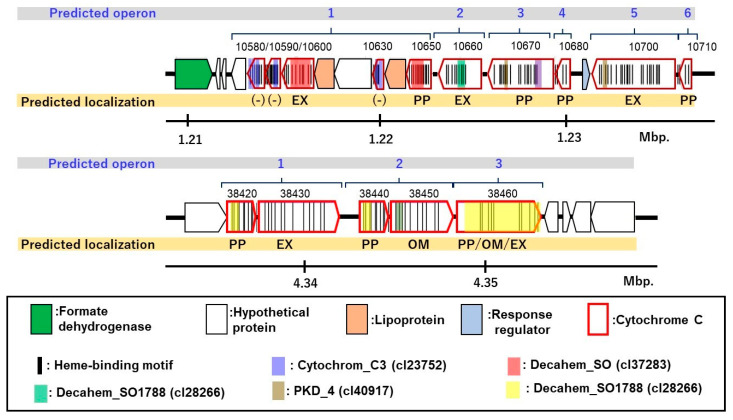
Two predicted gene clusters of unique *c*-type cytochrome C in strain NIT-T3.

The *c*-type cytochromes were highly variable showing approximately ~77.76% similarity to those of *Desulfuromonas* strains. The phylogeny matched within families of *Geobacteraceae* and *Desulfuromonadaceae*, and few were related to cytochromes found only in metagenomes. Among them, six *c*-type cytochromes were unique and shared <40% AAI with those in the database; four independent genes (DESUT3_0860, 20270, 29420, DESUT3_29000), and two (DESUT3_10660, 38460) were present in gene clusters of putative cytochromes (Figure 6).

### 3.7. Homologs of c-Type Cytochromes to Those in G. sulfurreducens PCA

Twenty-four *c*-type cytochromes were homologs of those functionally identified in *G. sulfurreducens* PCA. DESUT3_09000, 18560, and 27920 are homologous to PpcD, DESUT3_10670 and 38450 are homologous to OmcB, and DESUT3_40090 and 40040 are homologous to ImcH and CbcL present in *G. sulfurreducens*, respectively. These cytochromes are involved in the porin-cytochrome (Pcc) EET pathways that mediate electron transfer across the cell envelope [51]. In the Pcc pathways, ImcH and CbcL represent inner membrane cytochromes that oxidize quinol in the cytoplasmic membrane and transfer the released electrons to the periplasmic PpcA/PpcD [52,53]; PpcA/PpcD transfers the electrons acquired from the cytoplasm to the OmcB-based (ombB-omaB-omcB) conduit in the outer membrane [54]. The OmcB-based conduit transfers electrons through the lipid bilayer of proteoliposomes and directly reduces Fe(III) hydroxides outside the proteoliposomes [55,56]. 

DESUT3_29940, 36260, 05490, 10630, and 31560 are homologs of ExtA, ExtC, ExtG, ExtT, and ExtK of *G. sulfurreducens*, respectively. They belong to the outer membrane electron conduit *ext* gene clusters, which transfer electrons across the outer membrane to the bacterial surface [57]. DESUT3_31530 and 31540 are homologs of CbcM, and DESUT3_37260, 31490, 40060, and 12140 are homologs of CbcA, CbcR, CbcS, and CbcX of *G. sulfurreducens*, respectively. These Cbc-proteins and CbcL constitute the menaquinol oxidoreductase protein complexes, which combine electron transfer processes to form a proton gradient across the inner membrane via either a Q loop or a Q cycle [58,59]. 

DESUT3_40120 is a homolog of the inner-membrane-associated diheme cytochrome MacA of *G. sulfurreducens*, which is described as a peroxidase that can also mediate the electron transfer between inner membrane components and multiheme periplasmic cytochromes [60]. DESUT3_20370, 37510, and 29950 are homologs of the outer membrane cytochrome OmcI, OmcV, and OmcX of *G. sulfurreducens*, respectively. These Omc-proteins help transfer the electrons extracellularly, are required for Fe (III) reduction [61,62], and are significantly upregulated by Fe(III) oxides/Mn(IV) oxides/granular activated carbon [61,63]. DESUT3_40720 is a homolog of NrfA, which functions as a nitrite reductase component in a putative NrfH/NrfA nitrite-to-ammonia respiration pathway [25]. DESUT3_31280 is a homolog of CoxB encoding a cytochrome c oxidase subunit II, which is involved in the oxidative phosphorylation pathway. 

### 3.8. Type IV Pilus (T4P)-Related Genes

**Figure 7:**
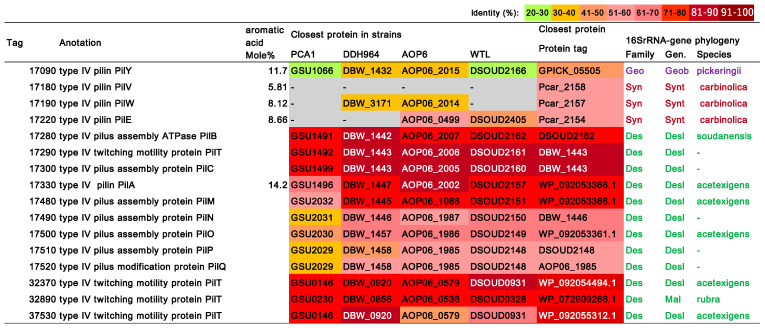
List of T4P assembly-related genes in the NIT-T3 genome Tag indicates the locus tag of coding sequences encoding T4P-related proteins in the genome. The number and color scale for the closest protein in the strain indicates the locus tag and amino acid identity (%), respectively. T4P, Type IV pilli; Geo, Geobacteraceae; Geob, *Geobacter;* Syn, *Syntrophotaleaceae;* Synt, *Syntrophotalea;* Des, *Desulfuromonadaceae;* Desl, *Desulfuromonas;* Mal, *Malonomonas*.

The strain NIT-T3 was found to contain 16 CDSs encoding T4P (Figure 7) and 3 related genes including transcription regulators. T4P are filamentous polymers of pilin monomers that undergo dynamic rapid polymerization and depolymerization from a pool of pilin [64]. The pilus polymer of pilin protein PilA of *G. sulfurreducens* PCA is an electrically conductive pilus and is known to be involved in the EET of solid electron acceptors [65]. Aromatic acids are key elements associated with conductivity and are estimated to account for 9.83% of the content in the PilA of strain PCA [66]. The aromatic acid content in the PilA homolog in NIT-T3 was 14.2%, which was relatively high based on the range of PilA aromatic acid content in phylogenetically diverse bacteria (5.5–25.25%) [67]; this suggested that the polymer may be conductive. The NIT-T3 genome was found to contain a full set of genes encoding T4P: two major pilins PilA and PilE, four minor pilins PilE, PilY, PilV, and PilW, and other essential proteins for the secretin (PilQ), alignment (PilM, PilN, PilO, and PilP), platform (PilC), four retraction ATPases (PilT), and assembly ATPases (PilB). Most CDSs are well-conserved in the genus *Desulfuromonas*, except for CDSs of four minor pilins closely related to the genera *Syntrophotalea* (PilE, PilV, and PilW) and *Geobacter* (PilY).

## 4. Discussion

The isolation of a novel electrogenic strain NIT-T3 of the genus *Desulfuromonas* and its respiration-ability specific to solid minerals suggests the adaptation of the genus to solid minerals or conductor-driven metabolism [8,9,10,11,12,13]. The ability to grow on solid minerals and electrodes is supported by the presence of seven extracellular *c*-type cytochromes (Figure 5), and conductive pilin (PilA)-homologs and full sets of T4P-assembly genes in the genome (Figure 6). These CDSs are phylogenetically cross-related in the order *Desulfuromonadales* beyond family, and show higher identities with CDSs of the genera *Desulfuromonas* and *Malonomonas* of the family *Desulfuromonadaceae; Geobacter*, *Geoalkalibacter*, and *Geopsychrobacter* of the family *Geobacteraceae*, and *Syntrophotalea* of family *Syntrophotaleaceae*. The fact that all members in these genera are capable of reducing solid minerals, and that the EET-related genes are closely related suggests the early divergence of EET during the evolution of bacteria in the order *Desulfuromonadales*. The strain NIT-T3 required NaCl and showed growth in the presence of 0.05–3.0% NaCl; the preference for NaCl was in agreement with the salt tolerance observed in the genus *Desulfuromonas* (0–3.0%) [17,19,21]. This supports the fact that *Desulfuromonas* species have widely adapted to both marine and freshwater environments.

The *c*-type cytochromes were hypervariable and abundant (n = 79), whereas other *Desulfuromonas* strains are reported to contain 37–44 *c*-type cytochromes according to the given annotation. The difference in the number was probably due to the difference in annotation strategies. A CDS for *c*-type cytochrome (DESUT3_5430) had 53 heme-binding sites which was the maximum number, and showed 54% of the closest identity to that of *Geoalkalibacter subterraneus* (B_0221) and 49% and 41% with that of contigs of *D. acetexigens* and *M. rubra*, respectively. The number of heme-binding sites in these homologs ranged from 55 to 77, indicating the broad distribution of the *c*-type cytochrome with such a large number of heme-binding sites in the order *Desulfuromonadales*.

Among the unique *c*-type cytochromes, two cytochromes (DESUT3_10660 and DESUT3_38460) were present in gene clusters including multiple *c*-type cytochromes (Figure 7). The gene cluster harboring DESUT3_10660 contains ten non-cytoplasmic *c*-type cytochromes with domains for cytochrome_C3 (cl23752), decahem_SO (cl37283), and decahem_SO1788 (cl28266). Three homologs of *c*-type cytochromes are found in strain PCA: DESUT3_10630 sharing 49% identity with ExtT which is a subunit of ExtTUVW conduit, and DESUT3_10680 and DESUT3_10710 with both sharing 43% identity with CtcA and tetra/tri-heme *c*-type cytochromes, respectively. DESUT3_38460 was located in a gene cluster including five CDSs of *c*-type cytochromes: three CDSs (DESUT3_38420, 38440, and 38450) shared 41–69% similarity with the *c*-type cytochrome of *Desulfuromonas* strains, and DESUT3_38430 showed 53% similarity with a hypothetical protein of *Desulfuromusa kysingii*. Among them, only one CDS (DESUT3_38450) was the homolog of the functionally identified *c*-type cytochrome known as omcB, a subunit of the OmaB/OmbB/OmcB conduit. The function of these gene clusters is unknown; however, it is speculated that they are essential for the unique adaptation of the strain for facilitating EET.

**Figure 8:**
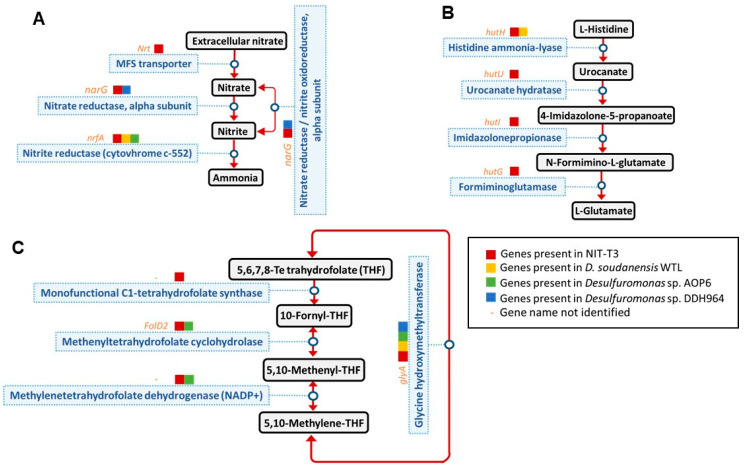
The complete metabolic pathway of strain NIT-T3 distinct from that of the complete genomes of three *Desulfuromonas* strains. Metabolic pathways of (**A**) nitrate reduction, (**B**) histidine degradation, and (**C**) C1-unit interconversion.

Strain NIT-T3 could metabolize nitrate which is not observed in other strains in the genus *Desulfuromonas*. The strain NIT-T3 produced nitrite; however, its production was approximately one-third of the amount of nitrate supplemented (Figure 3). The difference in the concentrations of produced nitrite and supplemented nitrate may be attributed to the assimilation of ammonia produced from nitrite. Analysis of the genome of NIT-T3 indicated the possibility of nitrite-to-ammonia transformation. Similar to NIT-T3, the complete genomes of all four *Desulfuromonas* strains contain CDSs for the nitrogen fixation pathway, including nitrite-to-ammonia reduction. However, genes encoding transporters for the uptake of extracellular nitrate were present in NIT-T3 alone (Figure 8A). This is consistent with the fact that NIT-T3 alone, and not the other *Desulfuromonas* strains, could reduce nitrate. 

The NIT-T3 genome was found to contain several unique CDSs that encode complete metabolic pathways which are absent in the other three *Desulfuromonas* genomes: histidine degradation to glutamate (Figure 8B), and C1-unit interconversion (Figure 8C). This suggested a variable metabolism in members of the genus *Desulfuromonas* associated with the transient reduction and uptake of genes in the genome.

In addition to the unique genes mentioned above, NIT-T3 possesses 46 other CDSs in the genome with no confirmed homologs (<40%) in other species of the *Desulfuromonas strains*; 29 of these sequences encode proteins with predicted functional annotation (Table S1). The unique proteins include a putative autoinducer-2 (AI-2) transporter family protein (DESUT3_07100), which has been reported to regulate the intracellular concentration of AI-2, a quorum-sensing chemical that affects global gene expression in biofilms [68,69] and shares a higher identity with proteins of *Geobacter* species than those of *Desulfuromonas*. The NIT-T3 genome includes another candidate for the AI-2 transporter family protein (DESUT3_31000) that was similar to that in all three genomes of the genus *Desulfuromonas,* but not to that of *Geobacter* species (63–70% identity). These two paralogs of AI-2 transporters with different phylogenies may potentially control biofilm growth. DESUT3_07210 encodes RNA molecules of a labile antitoxin, HicB, that regulates the expression of a toxin protein, HicA. Both proteins are involved in bacterial survival under stress conditions, such as nutrient deprivation and antibiotics [68,69]. An additional set of CDSs for HicAB was present in the genome; however, its role in *Desulfuromonadales* has never been investigated. Several metabolic genes (DESUT3_29720, 35500, 33690, and 37760) were also found to be unique to the NIT-T3 genome; however, these genes are disconnected from other functional genes and do not complete a series of metabolic processes.

## 5. Conclusions

The isolation and polyphasic characterization of the novel strain NIT-T3 revealed an increase in the metabolic capability of the genus *Desulfuromonas*. In total, 79 of the large number of *c*-type cytochromes suggested their substantial role as representative electrogenic bacteria in natural and synthetic environments. The interswitching phylogeny of EET-related genes in members of the order *Desulfuromonadales* beyond the family suggested the early divergence and the substantial roles of the order *Desulfuromonadales* rather than the specific genus like *Geobacter* in EETs in various environments. Strain NIT-T3 is proposed as a new species of the genus *Desulfuromonas* according to the phenotype and phylotype and the description is provided as follows:

### Description of Desulfuromonas versatilis sp. Nov.

*Desulfuromonas versatilis* (ver.sa’ti.lis L. masc./fem. Adj. versatilis, versatile with respect to the capability to use a variety of electron donors and acceptors). 

Cells are Gram-negative, non-spore-forming, rod-shaped, non-motile, and strictly anaerobic. Optimal growth was observed at 25 °C, pH 6.8–7.1, and NaCl concentration ranging from 0.2% to 1%. The substrates used for nitrate reduction included hydrogen, formate, acetate, lactate, pyruvate, succinate, malate, isopropanol, peptone, and yeast extract. Elemental sulfur, iron oxide, fumarate, nitrate, AQDS, malate, graphite electrode, and GO served as terminal electron acceptors coupled to acetate oxidation. The major cellular fatty acids present were C_16:1_ω7*c* and C_16:0_, and the major respiratory quinone in the cell wall of NIT-T3 was MK-8. The type strain, NIT-T3^T^ was isolated from a mixture of seawater and coastal sand. The genomic G + C content in the type strain was 63.1%.

## Figures and Tables

**Table 1:** Characteristics of strain NIT-T3 and other *Desulfuromonas* strains.

Characteristic	1	2	3	4	5	6	7	8	9	10
Motility	NM	ND	NM	Motile	Motile	NM	Motile	NM	Motile	Motile
G + C content (%)	63.1	61.2	61.2	50.1	ND	ND	61.6	54.7	62.3	62.3
Temp. range (°C) (Optimum)	10–35 (25)	24	10–37 (25–37)	2–20 (14)	10–35 (25)	21–31	10–39 (26–30)	40	(30–35)	(30–35)
pH range (Optimum)	6.4–8.4 (6.8–7.1) *	6.8	6.5–8.0 (7.0)	6.5–7.5 (7.3)	6.8–8.0 (7.0–7.5)	6.5–7.4 (7.4)	6.5–8.2 (6.9–7.9)	ND	6.4–8.5 (7.6–7.8)	6.4–8.5 (7.6–7.8)
Electron donors										
Hydrogen	+	+	(+)	-	-	-	-	(+)	-	-
Lactate	+	+	+	-	+	-	-	+	-	-
Fumarate	+	-	+	-	+	-	+	+	-	-
Succinate	+	-	+	-	+	-	+	+	-	-
Malate	+	ND	+	-	+	-	-	ND	-	-
Acetate	+	+	+	+	+	+	+	+	+	+
Pyruvate	+	+	+	+	+	+	+	-	ND	-
Glucose	-	-	-	-	-	ND	-	-	-	-
Butyrate	-	-	-	-	-	ND	-	-	-	-
Glycerol	-	-	ND	-	-	ND	-	ND	-	-
Peptone	+	ND	ND	ND	ND	ND	-	ND	ND	-
Isopropanol	+	ND	ND	ND	ND	ND	ND	ND	ND	ND
Ethanol	-	+	ND	+	-	-	-	-	-	+
Benzoate	-	-	-	ND	ND	-	-	-	-	-
Methanol	-	-	ND	ND	-	ND	-	-	-	-
Phenol	-	ND	-	ND	ND	ND	ND	-	ND	ND
Fructose	-	ND	-	-	-	ND	-	-	-	-
Isobutyrate	-	ND	ND	ND	ND	ND	ND	ND	ND	ND
Caproate	-	ND	ND	ND	ND	ND	-	ND	-	ND
Butanol	-	ND	ND	+	ND	ND	-	ND	-	+
Fermentation of	Fumarate	ND	Fumarate	ND	Fumarate malate	None	ND	ND	ND	ND
Electron acceptors										
Nitrate	+	ND	-	-	-	-	-	-	-	-
Sulfur	+	ND	+	+	+	ND	+	+	+	+
Sulfate	-	ND	-	-	-	-	-	-	-	-
Thiosulfate	-	ND	-	-	-	-	-	-	-	-
Ferric iron (Fe(III))	+	ND	+	+	+	+	(+)	+	ND	ND
Malate	+	ND	-	-	+	-	-	-	+	+
Fumarate	ND	ND	-	+	+	+	-	+	+	+
AQDS	+	ND	-	ND	ND	ND	ND	ND	ND	ND
GO	+	ND	ND	ND	ND	ND	ND	ND	ND	ND
Major fatty acids (>10%)	C_16: 1ω7c_ (26.2%) C_16: 0_ (18.3%) C_15: 0_ (13.2)	ND	C_16: 0_ (39.3%) C_16:1ω7c_ and/or iso-C_15:0_ 2-OH (36.6%)	C_16: 0_ (43%) C_16:1_ω7*c* (35%) C_15: 0_ (10%)	ND	ND	ND	ND	ND	ND
Major respiratory quinones	MK-8 (93%) MK-9 (5.3%) MK-7 (1.9%)	ND	ND	ND	ND	ND	ND	ND	ND	ND
Strains: 1, NIT-T3 (this study); 2, *Desulfuromonas soudanensis* WTL^T^ [21]; 3, *Desulfuromonas carbonis* ICBM^T^ [18]; 4, *Desulfuromonas svalbardensis* 112^T^ [17]; 5, *Desulfuromonas michiganensis* BB1^T^ [16]; 6, *Desulfuromonas chloroethenica* TT4B^T^ [15]; 7, *Desulfuromonas thiophila* NZ27^T^ [14]; 8, *Desulfuromonas palmitatis* SDBY1^T^ [13]; 9, *Desulfuromonas acetexigens* 2873^T^ [12]; 10, *Desulfuromonas acetoxidans* DSM 684^T^ [11]. The data for NIT-T3 was obtained in this study and others are brought from references [14,15,16,17,18,19,20,21].										
+, good growth; (+), hydrogen was oxidized but no growth; -, no growth; [motile], only a small population was motile; ND, not determined; NM, not motile; temp., temperature; AQDS, Anthraquinone-2,6-disulfonate; GO, graphene oxide.										

* Tested with acetate and fumarate as the substrates.

## Data Availability

The data presented in this study are available on request from the corresponding author.

## Supplementary Materials

The following are available online at https://www.mdpi.com/article/10.3390/microorganisms9091953/s1, Table S1: Genes uniquely present in strain T3 compared with other species of the genus *Desulfuromonas*.
