# Supplementary material for: Isolation and Polyphasic Characterization of Desulfuromonas versatilis sp. Nov., an Electrogenic Bacteria Capable of Versatile Metabolism Isolated from a Graphene Oxide-Reducing Enrichment Culture
Source: Microorganisms. 2021 Sep 14;9(9):1953. doi: 10.3390/microorganisms9091953 (PMC8465243; doi:10.3390/microorganisms9091953)
Supplement: Supplementary file 1 [file microorganisms-09-01953-s001.zip › microorganisms-1314475-supplementary.pdf]

Supplemental material

Isolation and polyphasic characterization of 'Desulfuromonas versatilis' sp. nov., an electrogenic bacteria capable of versatile metabolism isolated from a graphene oxide-reducing enrich-ment culture

Li Xie[1,†], Naoko Yoshida[1,*,†], Shun'ichi Ishi [2], Lingyu Meng[1]

[1]Department of Civil Engineering, Nagoya Institute of Technology (Nitech), Nagoya, Japan
[2] Institute for Extra-cutting-edge Science and Technology Avant-garde Research (X-star), Japan Agency for Marine-Earth Science and Technology (JAMSTEC), Yokosuka, Japan
[*]Correspondence: yoshida.naoko@nitech.ac.jp; Tel.: +81-527-355-437
[†]These authors have contributed equally to this work and share first authorship

Table S1 Genes uniquely present in strain T3 compared with other species of genus *Desulfuromonas*

Table S1 Genes uniquely present in strain T3 compared with other species of genus *Desulfuromonas*

Tag number	Annotation
DESUT3_01460	Putative Bsumi Modification Methylase Subunit Ydip
DESUT3_03580	Hypothetical Protein
DESUT3_05490	Hypothetical Protein
DESUT3_07100	Ai-2E Family Transporter
DESUT3_07130	Hypothetical Protein
DESUT3_07210	Antitoxin Hicb
DESUT3_07600	Hypothetical Protein
DESUT3_08270	Hypothetical Protein
DESUT3_12560	Hypothetical Protein
DESUT3_13100	Hypothetical Protein
DESUT3_13900	Lysr Family Transcriptional Regulator
DESUT3_13940	Antitoxin
DESUT3_15240	Hypothetical Protein
DESUT3_15390	Hypothetical Protein
DESUT3_15500	Cytochrome C
DESUT3_16990	Hypothetical Protein
DESUT3_17150	Hypothetical Protein
DESUT3_17920	Hypothetical Protein
DESUT3_17980	Hypothetical Protein
DESUT3_20410	Hypothetical Protein
DESUT3_20740	Lipoprotein
DESUT3_20860	Hypothetical Protein
DESUT3_22960	Hypothetical Protein
DESUT3_23860	Type I Restriction-Modification System Deoxyribonuclease
DESUT3_23880	Type I Restriction Endonuclease Ecoki Subunit S
DESUT3_26230	Hypothetical Protein
DESUT3_26440	Hypothetical Protein
DESUT3_27260	Hypothetical Protein
DESUT3_28600	Hypothetical Protein
DESUT3_29720	Glutathione S-Transferase
DESUT3_30960	Hypothetical Protein
DESUT3_31320	Hypothetical Protein
DESUT3_33270	Hypothetical Protein
DESUT3_33460	Flavodoxin Family Protein
DESUT3_33690	Alpha/Beta Hydrolase
DESUT3_33960	Hypothetical Protein
DESUT3_34150	Methyltransferase
DESUT3_34460	Hypothetical Protein
DESUT3_34990	Hypothetical Protein
DESUT3_35500	Molybdopterin-Guanine Dinucleotide Biosynthesis Protein Moba
DESUT3_37610	Hypothetical Protein
DESUT3_37660	Hypothetical Protein
DESUT3_37760	Gdp-L-Fucose Synthase
DESUT3_38080	Two-Component Sensor Histidine Kinase
DESUT3_38460	Cytochrome C
DESUT3_40760	Type Iv Pilin
DESUT3_40780	Hypothetical Protein
